# Systemic immunity-inflammation index and body mass index: A cross-sectional study

**DOI:** 10.1371/journal.pone.0327017

**Published:** 2025-07-16

**Authors:** Wei Guo, Chongheng Zhang, Fang Cao, Xinxin Sun, Jian Li, Wenfeng Zhang

**Affiliations:** 1 Changchun University of Chinese Medicine, Changchun, China; 2 Chinese People's Armed Police Force Characteristic Medical Center, Tianjin, China; State University of Rio de Janeiro, BRAZIL

## Abstract

**Background and aim:**

The Systemic Immunity-inflammation Index (SII) is an emerging metric for assessing an individual’s immunological and inflammatory condition. Studies examining the relationship between SII and body mass index (BMI) are limited. This study aims to clarify the correlation between SII and BMI.

**Methods:**

The current study investigated the association between SII and BMI using data from the National Health and Nutrition Examination Survey (NHANES). Information on SII and BMI was obtained from laboratory and demographic data. The analytical approach included techniques such as linear univariate analysis, linear multivariate analysis, and subgroup analysis to explore the intricate relationship between SII and BMI comprehensively.

**Results:**

A total of 1568 participants who met the inclusion criteria were included in the study; their average age was 53.6 ± 16.7 years, and they were 40.8% female and 59.2% male. Several baseline indicators showed statistically significant differences across SII groups. Curve fitting revealed an inverted L-shape, non-linear relationship between SII and BMI. Multivariate linear regression analysis revealed that adjusted β values were 1.7 (95% CI: 0.87–2.54, *p* < 0.001) for T3 and 0.46 (95% CI: −0.36–1.28, *p* = 0.271) for T2 in comparison to T1. Threshold analysis indicated that SII < 677.842 was associated with a BMI increase β of 0.005 (95% CI: 0.002–0.007, *p* < 0.001). The stratified analysis demonstrated the positive association between SII and BMI was not influenced by gender, diabetes, or smoking (all *p* > 0.05).

**Conclusions:**

In summary, these findings reveal a robust association between SII and BMI within the examined American population.

## 1. Introduction

The Systemic Immunity-Inflammation Index (SII) is an innovative inflammatory marker. The calculating method is SII platelet count multiplied by neutrophil count divided by lymphocyte count. The SII was initially introduced to assess the prognosis of patients with hepatocellular carcinoma following radical resection [1]. Afterward, it was applied in cancer research for pancreatic cancer [2]and prostate cancer [3]. SII has also been used to evaluate inflammation in the endocrine direction. In recent years, SII has been considered to be related to type 2 diabetes nephropathy [4], hyperlipidemia [5], diabetes foot [6], and non-alcoholic fatty liver [7]. SII has received significant attention and has been extensively studied for its capacity to assess inflammation and enable precise acquisition.

Body mass index (BMI) is a commonly used international measurement for assessing the extent of obesity and overall health of the human body. A BMI greater than 25 kg/m^2^ is classified as overweight, whereas a BMI exceeding 30 kg/m^2^ is classified as obese. Overweight and obesity constitute significant global health issues and are risk factors for numerous diseases, including diabetes and non-alcoholic fatty liver disease. Obesity can induce persistent systemic inflammation and may result in insulin resistance (IR), precipitating many illnesses. The correlation between SII and BMI warrants investigation as a recently established inflammatory marker. Clarifying the relationship between SII and BMI could help to reveal the state of metabolism-related inflammation and enhance public awareness of the adverse health consequences associated with obesity. Therefore, this study examined the correlation between SII and BMI prevalence in a cross-sectional design to further understand this area.

## 2. Materials and methods

### 2.1. Study population

**Fig 1:**
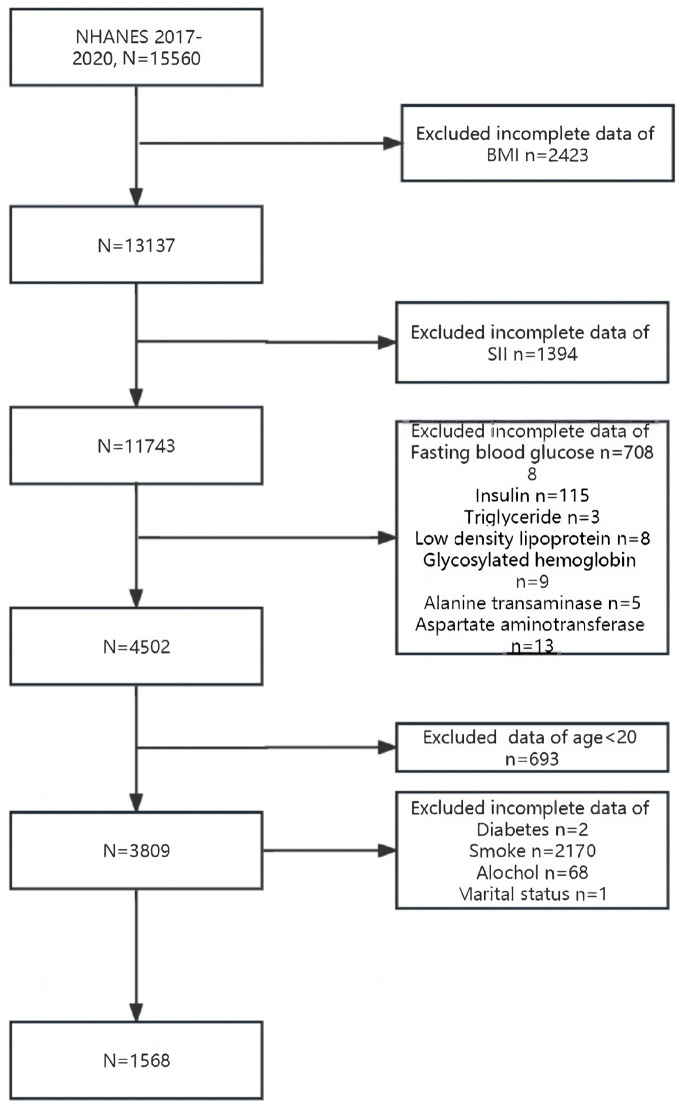
Participant selection flowchart. The specific participant screening and selection process is included in the flowchart.

The National Center for Health Statistics (NCHS) administers the National Health and Nutrition Examination Survey (NHANES) study, which assesses the health and nutritional status of the US population. The NHANES data is accessible at https://www.cdc.gov/nchs/nhanes/NHANES. A stratified multi-level probability sampling technique was incorporated into the research design to ensure that the samples were significantly representative. Data from the NHANES survey is available to the public every two years. Data for this cross-sectional study originated from surveyors who participated in NHANES between March 2017 and 2020. All participants in the NHANES study provided written consent, which was approved by the NCHS Research Ethics Review Committee. The acquired data was carefully cleansed to ensure the highest accuracy in the findings. Finally, 1568 responders satisfied the requirements. As shown in Fig 1.

### 2.2. Exposure and ending definition

This study regarded SII as an exposure variable, with SII data obtained from NHANES 2017–2020. The complete results of SII are derived from the platelet count multiplied by the ratio of neutrophil count to lymphocyte count. The outcome variable of this study is BMI. The comprehensive BMI assessment data are available on the NHANES website.

### 2.3. Covariates

Covariables that may affect the relationship between SII and BMI are also included in our survey. Continuous variables include age, total cholesterol (TC), triglycerides (TG), low-density lipoprotein cholesterol (LDL-C), high-density lipoprotein cholesterol (HDL-C), fasting plasma glucose (FPG), insulin (INS), alanine aminotransferase (ALT), aspartate aminotransferase (AST), and glycosylated hemoglobin (HbA1c). Classified variables include gender, race (Mexican American/other Hispanic/non-Hispanic black/non-Hispanic white/other race), marital status (married/divorced/never married), and education level (lower than grade 9/grade 9–11/high school or GED/some college or AA degree/college degree or above), diabetes (yes, no, border), smoking (daily, occasionally, never), and drinking (yes, no). These were derived from NHANES demographic data, screening information, questionnaire surveys, and laboratory assessments. Detailed measurement protocols are available on NHANES (https://www.cdc.gov/nchs/nhanes/).

### 2.4. Statistical analysis

All participants underwent a descriptive analysis. Whereas continuous data is represented by the mean ± standard deviation or median (interquartile spacing), classified data is represented by numbers (percentages). The continuous variables between the two groups were evaluated using the Kruskal-Wallis H test or analysis of variance. In comparison, Fisher’s exact or chi-square tests were used to evaluate the categorical variables between the two groups. Univariate and multivariate linear regression models were used to investigate the relationship between SII and BMI; regression coefficients (β) and 95% CI were obtained. These confounding variables were selected for the current study because of their relationship to the results of our attention. Adjusted by race and gender in Model 1; adjusted by race, smoking, diabetes, and gender in Model 2. Model 3 included adjustments for gender, race, diabetes, smoking, TC, TG, HDL-C, FPG, INS, AST, and HbA1c. Horizontal stratified subgroup investigations were conducted along with threshold analysis and smooth curve fitting to investigate the non-linear association between SII and BMI.

The statistical software program R (http://www.R-project.org, The R Foundation) and Free Statistical Software version 1.9 were used for all data analysis in the present study. The threshold for statistical significance is set at *p *< 0.05.

## 3. Results

### 3.1. Baseline information description

A total of 1568 people have sufficient data to meet the inclusion criteria for this study. The baseline characteristics of all subjects are described in Table 1. The study included 640 female and 928 male participants (59.2%). The participants are 53.6 ± 16.7 years old on average. Between SII groups, there were statistically significant mean or median differences in TC, LDL-C, INS, gender, age, race, marital status, ALT, AST, and diabetes (*p *< 0.05).

**Table 1:** Stratified the baseline characteristics of study participants based on the third percentile of SII.

Characteristic	SII				
	Total (n = 1568)	T1 (n = 523)	T2 (n = 522)	T3 (n = 523)	*P-*value
TC (mmol/l)	4.7 ± 1.1	4.7 ± 1.1	4.8 ± 1.1	4.6 ± 1.1	0.012
TG (mmol/l)	1.3 ± 0.9	1.2 ± 0.9	1.3 ± 0.9	1.3 ± 0.8	0.130
LDL-C (mmol/l)	2.8 ± 1.0	2.8 ± 1.0	2.9 ± 1.0	2.7 ± 0.9	0.025
HDL-C (mmol/l)	1.4 ± 0.4	1.4 ± 0.4	1.4 ± 0.4	1.3 ± 0.4	0.072
FPG (mmol/l)	6.5 ± 2.3	6.3 ± 2.2	6.5 ± 2.4	6.6 ± 2.4	0.091
INS (μmol/l)	15.0 ± 24.2	12.6 ± 13.5	15.1 ± 29.1	17.2 ± 26.9	0.009
Gender					0.001
Male	928 (59.2)	341 (65.2)	303 (58)	284 (54.3)	
Female	640 (40.8)	182 (34.8)	219 (42)	239 (45.7)	
Age (year)	53.6 ± 16.7	51.9 ± 16.4	53.4 ± 16.6	55.4 ± 16.8	0.003
Race					< 0.001
Mexican American	178 (11.4)	65 (12.4)	56 (10.7)	57 (10.9)	
Other Hispanic	126 (8.0)	36 (6.9)	49 (9.4)	41 (7.8)	
Non-Hispanic White	655 (41.8)	156 (29.8)	236 (45.2)	263 (50.3)	
Non-Hispanic Black	404 (25.8)	191 (36.5)	116 (22.2)	97 (18.5)	
Other Race – Including Multi-Racial	205 (13.1)	75 (14.3)	65 (12.5)	65 (12.4)	
Education level					0.507
Less than 9th grade	109 (7.0)	41 (7.8)	34 (6.5)	34 (6.5)	
9-11th grade	227 (14.5)	71 (13.6)	81 (15.5)	75 (14.3)	
High school graduate/GED or equivalent	434 (27.7)	139 (26.6)	134 (25.7)	161 (30.8)	
Some college or AA degree	538 (34.3)	175 (33.5)	190 (36.4)	173 (33.1)	
College graduate or above	260 (16.6)	97 (18.5)	83 (15.9)	80 (15.3)	
Marital status					0.011
Married/Living with Partner	894 (57.0)	311 (59.5)	297 (56.9)	286 (54.7)	
Widowed/Divorced/Separated	415 (26.5)	113 (21.6)	140 (26.8)	162 (31)	
Never married	259 (16.5)	99 (18.9)	85 (16.3)	75 (14.3)	
ALT (mmol/l)	22.9 ± 23.6	26.4 ± 35.7	22.2 ± 15.1	20.3 ± 12.3	< 0.001
AST (mmol/l)	22.6 ± 18.5	26.1 ± 28.6	21.5 ± 10.3	20.2 ± 9.3	< 0.001
HbA1c (%)	5.9 ± 1.3	5.9 ± 1.2	5.9 ± 1.3	6.0 ± 1.3	0.472
Diabetes					0.019
Yes	295 (18.8)	86 (16.4)	86 (16.5)	123 (23.5)	
No	1222 (77.9)	421 (80.5)	419 (80.3)	382 (73)	
Boundary	51 (3.3)	16 (3.1)	17 (3.3)	18 (3.4)	
Smoke					0.231
Every day	520 (33.2)	173 (33.1)	165 (31.6)	182 (34.8)	
Occasionally	152 (9.7)	59 (11.3)	41 (7.9)	52 (9.9)	
No smoke	896 (57.1)	291 (55.6)	316 (60.5)	289 (55.3)	
Alcohol					0.43
Yes	1545 (98.5)	515 (98.5)	512 (98.1)	518 (99)	
No	23 (1.5)	8 (1.5)	10 (1.9)	5 (1)	
BMI (kg/m^2^)	30.2 ± 7.6	29.0 ± 6.8	30.0 ± 7.5	31.6 ± 8.3	< 0.001

The data is expressed as the average or median of continuous variables, while categorical variables are expressed as percentages.

TC, total cholesterol; TG, triglycerides; LDL-C, low-density lipoprotein cholesterol; HDL-C, high-density lipoprotein cholesterol; FPG, fasting plasma glucose; INS, Insulin; ALT, alanine aminotransferase; AST, aspartate aminotransferase; HbA1c, glycosylated hemoglobin.

### 3.2. Relationship between SII and BMI

Table 2 displays the relationship between SII and BMI as determined by multiple linear regression analysis. SII and BMI positively correlated in the unadjusted model [β = 1.29 (95% CI:0.83–1.75]). SII and BMI continued to have a positive correlation [β = 1.22 (95% CI:0.76–1.68)] after controlling for gender and race. After adjusting for all covariates, the relationship between SII and BMI remained unchanged [β = 0.86 (95% CI:0.44–1.27)], which means that for every unit increase in SII, BMI increased by 0.86 kg/m². All differences were statistically significant.

**Table 2:** Multivariate linear regression analysis of the correlation between SII and BMI.

	N	Unadjusted		Model 1		Model 2		Model 3	
		β (95%CI)	P-value	β (95%CI)	P-value	β (95%CI)	P-value	β (95%CI)	P-value
T1	523	0 (Ref)		0 (Ref)		0 (Ref)		0 (Ref)	
T2	522	0.98 (0.06 ~ 1.89)	0.037	0.9 (−0.01 ~ 1.81)	0.054	0.87 (−0.02 ~ 1.76)	0.054	0.46 (−0.36 ~ 1.28)	0.271
T3	523	2.57 (1.66 ~ 3.49)	<0.001	2.44 (1.52 ~ 3.36)	<0.001	2.17 (1.27 ~ 3.07)	<0.001	1.7 (0.87 ~ 2.54)	<0.001
P for Trend	1568	1.29 (0.83 ~ 1.75)	<0.001	1.22 (0.76 ~ 1.68)	<0.001	1.09 (0.64 ~ 1.54)	<0.001	0.86 (0.44 ~ 1.27)	<0.001

Model 1: Adjust for gender and race.

Model 2: Adjust for Diabetes and Smoking in addition to model 1.

Model 3: Adjust for TC, TG, HDL-C, FPG, INS, AST, and HbA1c in addition to model 2.

TC, total cholesterol; TG, triglycerides; HDL-C, high-density lipoprotein cholesterol; FPG, fasting plasma glucose; INS, insulin; AST, aspartate aminotransferase; HbA1c, glycosylated hemoglobin.

### 3.3. Curve fitting and threshold analysis

**Fig 2:**
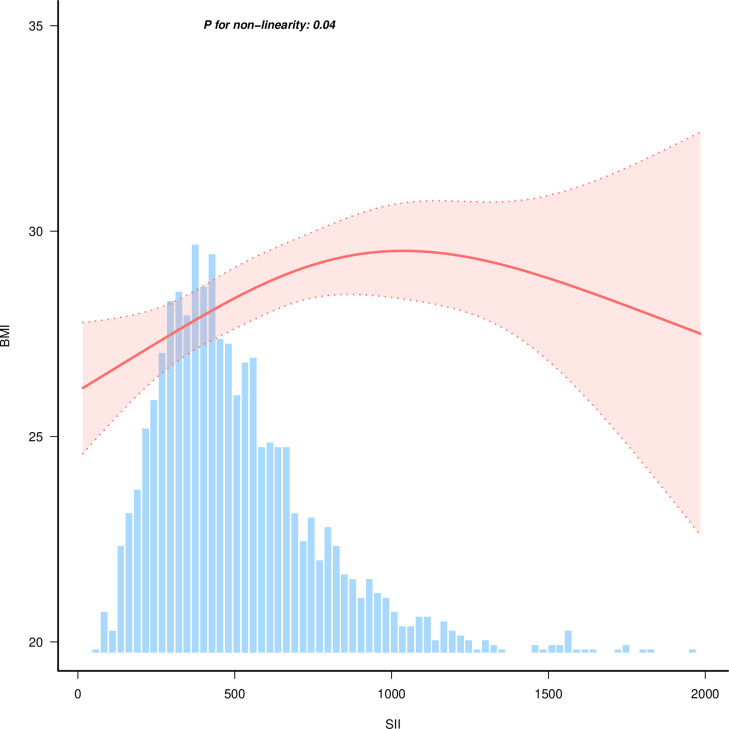
Curve fitting of Sll and BMI. Adjust for gender, race, Diabetes, Smoking, TC, TG, HDL-C, FPG, INS, AST and HbAlc. TC, total cholesterol; TG, triglycerides; HDL-C, high-density lipoprotein cholesterol; FPG, fasting plasma glucose; INS, insulin; AST, aspartate aminotransferase; HbAlc, glycosylated hemoglobin.

Furthermore, a non-linear association between SII and BMI was revealed by performing a curve fitting of SII to BMI (*p* = 0.04). As shown in Fig 2. The projected values and 95% CIs are shown in Fig 2 by the solid and dotted lines, respectively. Then, a threshold analysis based on the curve-fitting results was carried out. The results of the study showed a positive correlation between SII and BMI when SII < 677.842 [β = 0.005 (95% CI:0.002–0.007), *p* < 0.001]. When SII ≥ 677.842, there was no significant correlation between SII and BMI (Table 3). From this, we can determine that SII is associated with BMI in an inverted L-shape.

**Table 3:** Threshold effect analysis of the relationship of SII with BMI.

SII	Model	
	β (95% CI)	P-value
<677.842	0.005 (0.002-0.007)	<0.001
≥677.842	−0.001 (−0.004-0.001)	0.396
Likelihood Ratio test		0.003

Adjust for gender, race, Diabetes, Smoking TC, TG, HDL-C, FPG, INS, AST and HbA1c.

TC, total cholesterol; TG, triglycerides; HDL-C, high-density lipoprotein cholesterol; FPG, fasting plasma glucose; INS, insulin; AST, aspartate aminotransferase; HbA1c, glycosylated hemoglobin;SII, systemic immune-inflammation index; BMI, body mass index; β, regression coefficients; CI, confidence intervals.

### 3.4. Stratified analysis

**Fig 3:**
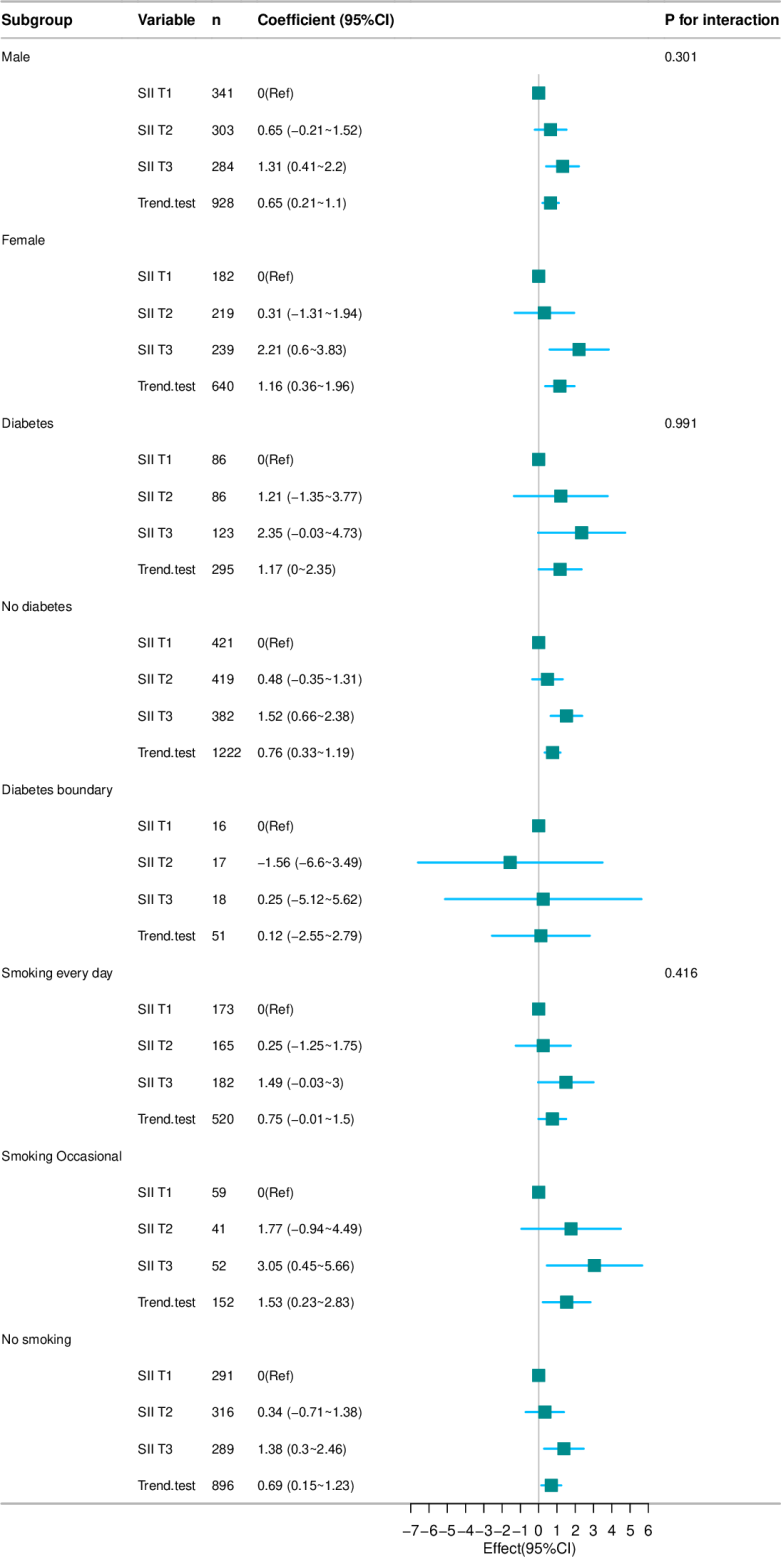
Subgroup analysis of SlI and BMI. Adjust for gender, race, Diabetes, Smoking, TC, TG, HDL-C, FPG, INS, AST and HbAlc. TC, total cholesterol; TG, triglycerides; HDL-C, high-density lipoprotein cholesterol; FPG, fasting plasma glucose; INS, insulin; AST, aspartate aminotransferase; HbAlc, glycosylated hemoglobin.

Furthermore, stratified analyses were conducted to determine if the positive correlation between SII and BMI was affected by gender, diabetes, or smoking. As shown in Fig 3. Statistical significance was observed in the subgroups of males, females, non-diabetics, occasional smokers, and non-smokers when evaluated by gender, diabetes status, and smoking behaviors (*p* < 0.05). In contrast, no statistically significant associations were observed in the subgroups with diabetes, on the verge of diabetes, and daily smoking (*p* > 0.05). However, the positive association between SII and BMI was not influenced by gender, diabetes, or smoking.

## 4. Discussion

The findings from a cross-sectional analysis of NHANES health screening participants indicated that BMI escalated by 0.86 kg/m² for each unit increase in SII. An inverted L-shaped correlation was observed between SII and BMI, with an inflection point at approximately 677.842; specifically, when SII was less than 677.842, SII and BMI exhibited a positive correlation, whereas when SII was equal to or greater than 677.842, SII and BMI were not statistically different. The stratified analysis showed that gender, diabetes, and smoking did not affect the relationship between SII and BMI.

Recent years have witnessed more clinical attention given to the health consequences of obesity, which has prompted researchers to investigate the adverse effects of obesity, so occupying more hotspots. From 1975 to 2014, adult obesity (BMI ≥ 30 kg/m^2^) has a prevalence rate [8]. The mortality rate of diseases caused by a high BMI similarly rises. The mortality rate from high BMI rose 265% in 2019 compared to 1990 [9]. Obesity now poses a major worldwide health concern.

The association between SII and BMI demands further study as a recently identified inflammatory marker. In his research, Wencong Guo observed that elevated SII levels correlated with diabetic nephropathy (DKD) in individuals with T2DM and suggested that SII could be a cost-effective and direct approach to DKD detection [4]. Similarly, Jiahang Li found that SII correlated with diabetic retinopathy (DR) and pointed out that SII has a high value in diabetic microvascular diagnosis [10]. This also corroborates the findings of Wencong Guo. Furthermore, Ke Liu et al. presented findings from a cohort of US adults, indicating a substantial correlation between SII and the likelihood of developing NAFLD, highlighting SII’s significance in predicting NAFLD risk [11]. These clinical trials validate a strong correlation between SII and metabolic disorders. SII offers insights into the inflammation associated with metabolic disorders and aids in monitoring treatment efficacy [12]. The current study of SII and BMI facilitates the examination of the correlation between inflammation and obesity, thereby enhancing our understanding of SII’s role in metabolic disorders, reinforcing the awareness of obesity’s hazards, and offering improved direction for future experiments and obesity research. Several investigations have been conducted on SII. The crucial value of SII remains unstandardized. BMI serves as an essential baseline metric, elucidating the correlation between BMI and SII, thus aiding in standardizing the key value for SII development.

Chronic inflammation is one of the primary research variables in obesity. These inflammations generally occur in insulin-target tissues, such as fat. TNF in obese adipose tissue: α Elevated expression and activation of IKK β enhance the production of inflammatory mediators [13]; simultaneously, macrophages within adipose tissue accumulate and demonstrate M1 polarization, leading to inflammation in adipose tissue and instigating systemic insulin resistance [14]. Theresa V. Rohm et al. believe that inflammation of insulin target tissues is a specific manifestation of immune metabolism [15]. The relationship between immune cells and metabolism throughout their differentiation and function is known as immune metabolism. Therefore, weight changes can also result from inflammation. NF-κB concerning age weight gain results from excessive NLRP-3 inflammasome activation brought on by B [16].

Moreover, NLRP-3 inflammasomes may also impair insulin signaling [17]. The correlation between inflammation and obesity has also been established in mice models. Damage to hypothalamus autophagy in mice activates hypothalamic IKK β, resulting in insulin resistance and obesity [18]. The relationship between endocrine metabolism and immunity is gradually established, but further research is still needed.

The SII is an index score derived from the combined measurements of lymphocytes, neutrophils, and platelets. The SII can assess the immunological and inflammatory condition of the body. An increase in SII indicates a higher inflammatory status and a decreased immunological status in the body. Neutrophils are a requisite for calculating the SII and constitute the most prevalent immune cells in the human body. They are intimately connected to obesity. Research indicates a substantial association between neutrophil count and elevated BMI [19]; also, neutrophil count reduces following surgical weight loss [20]. Eileen Uribe Querol summarized in a review that obese adipose tissue promotes systemic inflammation and leads to neutrophil production, activation, and proliferation [21].

During inflammation in adipose tissue, neutrophils augment the production of reactive oxygen species (ROS) and secrete TNF-α and IL-1β upon reaching the inflammatory site. Cytokines and chemokines modulate inflammation and stimulate lymphocytes to engage in ensuing inflammatory responses [22]. Lymphocytes comprise T cells, B cells, and natural killer (NK) cells. B cells were shown to be detrimental to obesity by Harry Kane et al. According to their animal studies, B cells in the adipose tissue of obese mice stimulated the production of neutrophil and T cell chemokines. They were more inflammatory than those in lean mice [23]. B cells encourage T cell activation by accumulating before T cells [24,25]. Based on surface differentiation antigens, T cells can be further classified into two subgroups, CD4 and CD8, which are crucial for the body’s immunological function. Pro-inflammatory Th1 and Th17, anti-inflammatory Th2, and regulatory T cells (Tregs) are the primary types of CD4 cells. Obesity reduces the number of CD4 Th2 cells.

CD4 Th1 cells, on the other hand, produce more IFN-γ. By releasing pro-inflammatory cytokines, CD8 cytotoxic T lymphocytes induce inflammation in adipose tissue [26]. Inflammation and obesity have also been studied using the neutrophil-to-lymphocyte ratio (NLR). Both viral and non-infectious immune responses can be detected by NLR [27]. NLR and abdominal obesity were significantly correlated in the study by Suárez Cuenca J.A. et al. [28]. A high NLR is a reliable indicator of type 2 diabetes [29]. Through mice experiments, Kain V. et al. proposed that the gut microbiota’s effect on white blood cell count could cause obesity’s impact on NLR [30]. Osadnik T.’s study findings could be explained by Kain V’s research findings: Some overweight people who appear healthy can have systemic inflammation detected by NLR [31]. In addition to being strongly related to SII, platelets are one of the conditions used to calculate SII. Obesity may be linked to elevated platelet counts, according to Johanna C. Purdy et al. [32]. Increasing vascular permeability to immune cells and immune cells’ ability to eliminate pathogens are important aspects of the immunological response, which includes inflammation [33]. Platelet activation and inflammation share several functional similarities, and platelets contribute to inflammation by releasing various small molecules and protein cytokines [34]. Following a clinical study, Sean P. Heffron also concluded that weight loss surgery can somewhat mitigate the effects of obesity, which is linked to alterations in the platelet transcriptome and enhanced platelet activation [35].

SII has several potential applications and is frequently employed in studying different disorders. Its benefits include cost-effectiveness and simplicity of use. There are advantages to the current research. The confounding factors were first corrected to increase the validity of the results. Second, a sufficient sample size was obtained. However, this study has some limitations. The current study is cross-sectional and cannot evaluate the causal link between exposure factors and outcomes. Furthermore, although the study’s confounding variables were controlled for, other confounding variables—like blood pressure—were not examined. Finally, this study does not include direct inflammatory markers (C-reactive protein, interleukins) to corroborate our findings, which is also one of our limitations. Therefore, further study is required to elucidate the connection between SII and BMI.

## Supporting information

S1 Table Single factor analysis of BMI. TC, total cholesterol; TG, triglycerides; LDL-C, low-density lipoprotein cholesterol; HDL-C, high-density lipoprotein cholesterol; FPG, fasting plasma glucose; INS, Insulin; ALT, alanine aminotransferase; AST, aspartate aminotransferase; HbA1c, glycosylated hemoglobin; SII, Systemic Immune-Inflammatory Index.
(DOCX)

S1 File Minimal data set. (CSV)
