# Supplementary material for: Systemic immunity-inflammation index and body mass index: A cross-sectional study
Source: PLoS One. 2025 Jul 16;20(7):e0327017. doi: 10.1371/journal.pone.0327017 (PMC12266463; doi:10.1371/journal.pone.0327017)
Supplement: S1 Table — TC, total cholesterol; TG, triglycerides; LDL-C, low-density lipoprotein cholesterol; HDL-C, high-density lipoprotein cholesterol; FPG, fasting plasma glucose; INS, Insulin; ALT, alanine aminotransferase; AST, aspartate aminotransferase; HbA1c, glycosylated hemoglobin; SII, Systemic Immune-Inflammatory Index. (DOCX) [file pone.0327017.s001.docx]

**Supplementary Table 1 Single factor analysis of BMI**

| Item | Coeff.(95%CI) | *P* |
| --- | --- | --- |
| TC(mmol/l) | -0.52 (-0.86,-0.18) | 0.003 |
| TG(mmol/l) | 1.6 (1.16,2.03) | < 0.001 |
| LDL-C(mmol/l) | -0.13 (-0.52,0.27) | 0.53 |
| HDL-C(mmol/l) | -4.92 (-5.74,-4.09) | < 0.001 |
| FPG(mmol/l) | 0.78 (0.62,0.94) | < 0.001 |
| INS(μmol/l) | 0.08 (0.07,0.1) | < 0.001 |
| Gender | 2.92 (2.17,3.68) | < 0.001 |
| Age (year) | -0.01 (-0.03,0.01) | 0.404 |
| Race: Mexican American |  | 0.036 |
| Other Hispanic | -0.2 (-1.94,1.54) |  |
| Non-Hispanic White | -0.94 (-2.2,0.33) |  |
| Non-Hispanic Black | -0.45 (-1.8,0.89) |  |
| Other Race - Including Multi-Racial | -2.15 (-3.69,-0.62) |  |
| Education level: Less than 9th grade |  | 0.186 |
| 9-11th grade | 0.71 (-1.03,2.45) |  |
| High school graduate/GED or equivalent | 0.87 (-0.73,2.47) |  |
| Some college or AA degree | 1.16 (-0.41,2.74) |  |
| College graduate or above | -0.13 (-1.84,1.58) |  |
| Marital status: Married/Living with Partner |  | 0.406 |
| Widowed/Divorced/Separated | -0.08 (-0.96,0.81) |  |
| Never married | 0.67 (-0.39,1.72) |  |
| ALT(mmol/l) | 0.01 (-0.01,0.02) | 0.349 |
| AST(mmol/l) | -0.03 (-0.05,-0.01) | < 0.001 |
| HbA1c (%) | 1.45 (1.16,1.74) | < 0.001 |
| Smoke: Every day |  | < 0.001 |
| Occasionally | 1.5 (0.13,2.87) |  |
| No smoke | 1.79 (0.97,2.61) |  |
| Alcohol | -2.42 (-5.56,0.73) | 0.132 |
| Diabetes: Yes |  | < 0.001 |
| No | -4.35 (-5.29,-3.4) |  |
| Boundary | -1.6 (-3.81,0.61) |  |
| SII: T1 |  | < 0.001 |
| T2 | 0.98 (0.06,1.9) |  |
| T3 | 2.57 (1.66,3.49) |  |

TC, total cholesterol; TG, triglycerides; LDL-C, low-density lipoprotein cholesterol; HDL-C, high-density lipoprotein cholesterol; FPG, fasting plasma glucose; INS, Insulin; ALT, alanine aminotransferase; AST, aspartate aminotransferase; HbA1c, glycosylated hemoglobin; SII, Systemic Immune-Inflammatory Index.
